# Theta–Beta/Gamma Coupling Identifies Bothersome Tinnitus Induced by Thalamocortical Dysrhythmia

**DOI:** 10.1002/brb3.70437

**Published:** 2025-06-12

**Authors:** Ying Wang, Jiajia Zhang, Xuan Huang, Shujian Huang, Yanmei Feng, Haibo Shi, Hui Wang, Richard Salvi, Shankai Yin

**Affiliations:** ^1^ Department of Otolaryngology‐Head and Neck Surgery Shanghai Sixth People's Hospital Affiliated to Shanghai Jiao Tong University School of Medicine Shanghai China; ^2^ Otolaryngology Institute of Shanghai Jiao Tong University Shanghai China; ^3^ Shanghai Key Laboratory of Sleep Disordered Breathing Shanghai China; ^4^ SUNY Distinguished Professor Center for Hearing and Deafness, 137 Cary Hall University at Buffalo New York USA

## Abstract

**Background:**

The phantom sound of tinnitus can be an extremely debilitating condition. The thalamocortical dysrhythmia (TCD) hypothesis is a feature of subjective tinnitus; however, its consistency in characterizing different types of tinnitus remains unclear.

**Method:**

We compared theta–beta/gamma coupling in multichannel EEG recordings from subjects with bothersome tinnitus (BT), non‐bothersome tinnitus (NBT), and healthy controls (HC). Additionally, we used these EEG features to distinguish BT from NBT by employing the k‐nearest neighbor (KNN) model.

**Results:**

Theta–beta/gamma phase‐amplitude coupling (PAC) was enhanced in the auditory cortex of both BT and NBT groups compared to HC. In contrast, theta–beta/gamma PAC was specifically enhanced in the cingulate gyrus and parahippocampal gyrus in the BT group. Notably, theta–gamma PAC in the orbitofrontal cortex was attenuated in the BT group and showed a negative correlation with their THI scores. By integrating theta–beta/gamma PAC into a machine learning algorithm, up to 92% of BT patients were accurately identified.

**Conclusion:**

TCD in BT patients was characterized by enhanced theta–beta/gamma PAC in the auditory cortex, cingulate gyrus, and parahippocampal gyrus and attenuated theta–gamma PAC in the orbitofrontal cortex. The latter was significantly negatively correlated with THI scores. These PAC features, which could objectively distinguish BT patients by machine learning, may support the specificity of PAC features in tinnitus characterization.

## Graphical abstract

The thalamocortical dysrhythmia (TCD) was observed in all patients with tinnitus, as evidenced by elevated PAC in the auditory cortex. Particularly, BT patients show characteristic theta–gamma PAC attenuation in the orbitofrontal cortex.

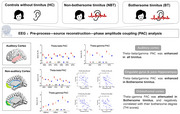

## 1 Introduction

Subjective tinnitus, a phantom auditory sensation (Baguley et al. 2013), affects approximately 447 million people worldwide, and its prevalence is expected to increase due to an aging population and greater exposure to noise (Rauschecker et al. 2010). Among individuals over 18 years of age, the prevalence of tinnitus ranges from 9% to 35% (McCormack et al. 2016), with 1–2% experiencing severe tinnitus that significantly disrupts daily life (Brinkmann et al. 2021), leading to negative social and economic consequences (Maes et al. 2013). One puzzling aspect of tinnitus is its heterogeneity: some patients are not bothered by their tinnitus, while others report devastating effects such as reduced quality of life, depression, anxiety, sleep disturbances, concentration difficulties, and impaired cognitive efficiency (Langguth 2011; Hallam et al. 2004). These severe symptoms are referred to as bothersome (BT) (Tunkel et al. 2014) or decompensated (Leńarz 1998) tinnitus. In extreme cases of BT, patients may even consider suicide (Lewis et al. 1994; Szibor et al. 2019), requiring prompt identification and intervention. However, effective methods for objectively distinguishing between severe cases of BT, which require therapeutic intervention, and NBT remain elusive.

Tinnitus is generally accompanied by peripheral auditory deafferentation caused by cochlear hearing loss. However, it can also occur in individuals without significant hearing loss (Weisz et al. 2006), potentially due to selective damage to inner hair cells or afferent auditory nerve synapses (Salvi et al. 2016; Liberman 2015). This cochlear deafferentation may lead to aberrant neural activity in the auditory system (Rauschecker et al. 2010; De Ridder et al. 2015; Vanneste et al. 2018), including neuronal hyperactivity (such as an enhanced spontaneous firing rate, SFR) or hypoactivity (a decreased SFR) (Seki and Eggermont 2003), and alterations in neuronal transfer functions (gain), tonotopic organization, and neural synchrony (Henton and Tzounopoulos 2021). Noise exposure has been reported to increase the SFR and neural synchrony of fusiform cells in the dorsal cochlear nucleus (DCN) and inferior colliculus (IC) (Niu et al. 2013). One potential mechanism underlying this increased neural activity is the long‐term potentiation of some synapses (Noreña 2011). Interestingly, using bimodal stimulation to reduce long‐term depression (LTD) has been demonstrated to alleviate physiological and behavioral evidence of tinnitus in both animals and humans (Marks et al. 2018). A recent study reported that reducing DCN neuronal cell activity in mice exhibiting noise‐induced tinnitus‐related behavior could diminish such behavior (Malfatti et al. 2022). However, lowering DCN activity during noise exposure did not prevent the onset of noise‐induced tinnitus, suggesting that this hyperactivity might not be critical for tinnitus induction but maintaining (Malfatti et al. 2022).

The medial geniculate body (MGB) is well‐positioned to play a critical role in tinnitus‐related auditory pathology due to its extensive connectivity (Henton and Tzounopoulos 2021). Peripheral auditory deafferentation disrupts the normal firing patterns of thalamic neurons, and studies in tinnitus mice have shown increased levels of tonic inhibition in the MGB (Sametsky et al. 2015). This altered activity is thought to initiate thalamocortical oscillations associated with tinnitus, as described by the thalamocortical dysrhythmia hypothesis (TCD) (De Ridder et al. 2014). Neural oscillations described in previous studies align with TCD‐related oscillations, such as increased gamma wave activity, which has been linked to tinnitus conditions (Weisz et al. 2007; Tzounopoulos et al. 2019). Notably, auditory cortex stimulation targeting regions of theta–gamma hyperactivity through implanted electrodes has been shown to suppress tinnitus. When such stimulation effectively reduces tinnitus, frequency spectral activities normalized both on electrode and source‐localized electroencephalography (EEG) recordings (De Ridder et al. 2011). Some researchers regard TCD as the neural substrate of tinnitus (Llinas et al. 2005), however, the precise mechanisms underlying TCD in tinnitus remain unclear.

Tinnitus is highly comorbid with negative emotions, and non‐auditory brain structures play a crucial role in the perception, maintenance, and severity of tinnitus in humans (Niu et al. 2013). Researchers have hypothesized that dysfunction in the limbic system, particularly in the prefrontal cortex and the striatal nucleus of the accumbens (NAc), may contribute to the generation of tinnitus perception (Rauschecker et al. 2010; Rauschecker et al. 2015). This idea is further supported by the noise‐cancellation model, which posits that limbic system dysfunction plays a causal role in the emergence of tinnitus perception (Leaver et al. 2011). According to the TCD model, the first necessary (but not sufficient) condition for tinnitus is sensory deafferentation, which triggers neuroplastic reorganization. This leads to hyperactivity and perceptual filling‐in of the deafferented frequency range, generating an imperceptible tinnitus signal that is suppressed by the limbic system. However, if the limbic region fails to function properly, the tinnitus signal can permeate to the auditory cortex, resulting in the perceptual emergence of tinnitus accompanied by auditory cortical reorganization (Rauschecker et al. 2010). Chronic stress is believed to be a key factor contributing to the perceptual emergence of severe tinnitus. When combined with hearing loss, chronic stress can lead to mild or severe disruption of the noise‐cancelation network, resulting in NBT or BT, respectively. Consistent with this view, tinnitus annoyance scores have been correlated with the degree of functional connectivity between auditory and stress‐related brain areas (Shore et al. 2016). In BT, increased synchrony has been observed between auditory areas and limbic regions associated with emotion, such as the subcallosal anterior cingulate cortex (scACC), parahippocampal gyrus, precuneus/posterior cingulate cortex (PCC), and dorsolateral prefrontal cortex. This enhanced synchrony between auditory‐limbic regions is associated with increased tinnitus‐related annoyance (Vanneste et al. 2010).

Analysis of TCD‐related oscillatory activity using machine learning has revealed stronger cross‐frequency coupling (theta–beta and theta–gamma) in the auditory cortex of tinnitus patients compared to healthy individuals (Vanneste et al. 2018). Given the significant role of the limbic system in tinnitus, we hypothesized that in individuals with tinnitus, the tinnitus signal propagates to the thalamus, leading to enhanced theta–beta/gamma coupling between the auditory cortex, limbic system (including the prefrontal cortex), and other brain regions. We further speculated that the strongest theta–beta/gamma coupling would occur in individuals with severe BT. To test this hypothesis, we employed source‐localized, resting‐state EEG to evaluate the theta–beta/gamma band coupling across the thalamus, auditory cortex, limbic system, and other regions of interest analysis (ROIs) in patients categorized as having high versus low tinnitus distress. Machine learning was subsequently used to validate these findings.

## 2 Methods

### 2.1 Study Design and Subjects

**FIGURE 1:**
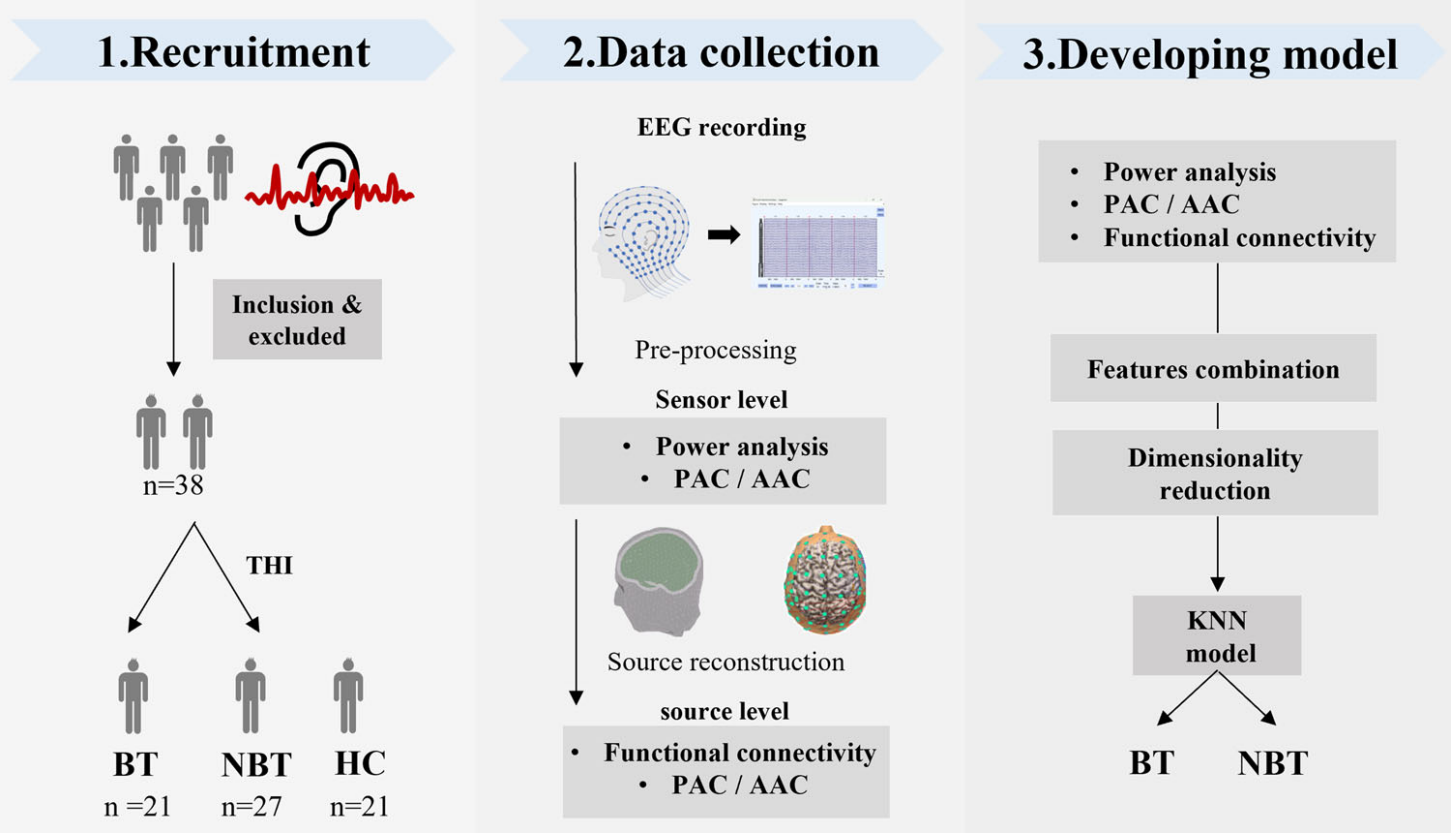
Schema of the data collection and analysis protocol.

Forty‐eight adults aged 22 to 76 years were recruited from the otolaryngology clinic of Shanghai Sixth People's Hospital Affiliated to Shanghai Jiao Tong University School of Medicine between January 2019 and October 2021. All participants were right‐handed, capable of following instructions, and had a clear understanding of the study's purpose. The inclusion criteria were as follows: (1) presence of subjective tinnitus symptomatic at the time of evaluation; (2) ability to complete the required tests; and (3) chronic tinnitus lasting more than 6 months. Exclusion criteria included: (1) cranial‐nerve‐related tinnitus, objective tinnitus, or pulsatile tinnitus; (2) conditions such as otosclerosis, Meniere's disease, or other local otitis diseases that could cause tinnitus or hyperacusis; (3) severe systemic disease (e.g., cardiac, hepatic, or renal failure); (4) neurological disorders such as brain tumors, traumatic brain injury, or stroke; and (5) psychiatric conditions (e.g., depression, anxiety, mania, and schizophrenia) caused by other diseases, and individuals receiving treatment for mental disorders. Participants were permitted or required to withdraw from the study if they experienced insurmountable claustrophobia, other intolerable conditions, major protocol violations, or the development of serious systemic diseases that precluded continued participation. Additionally, age‐ and gender‐matched volunteers with some degree of hearing loss but no tinnitus was recruited as healthy controls. The schema of the data collection and treatment protocol is illustrated in Figure 1.

This study was approved by the Institutional Ethics Review Board of Shanghai, the Sixth People's Hospital affiliated with Shanghai Jiao Tong University (Approval number: 2020–122), and registered with the Chinese Clinical Trial Registry (Number: ChiCTR2000035797).

### 2.2 Auditory Testing

We conducted the audiometric assessment, tympanogram, and distortion product otoacoustic emissions (DPOAEs) measurements. All auditory tests were performed by trained healthcare professionals in a soundproof booth. Audiograms were measured in 1‐octave steps at frequencies ranging from 0.25 to 8 kHz using a manual audiometer (GSI‐61, Grason‐Stadler Inc., Eden Prairie, MN, USA) equipped with TDH–39 headphones. DPOAEs were recorded using an otoacoustic emissions (OAE) system from Otometrics (Madsen Capella2, Natus Medical Denmark ApS, Taastrup, Denmark) with insert‐mounted probes. The assessments were performed in a soundproof room (< 30 dBA). Emissions were measured at 2*f*1–*f*2 frequencies with *f*2 = 2, 3, 4, 6, and 8 kHz. The *f*2/*f*1 ratio was set to 1.2. Response growth functions were collected using L2 levels from 35 to 85 dB SPL measured in 5 dB steps. The level of L2 was 10 dB less than that of L1. Tympanograms were obtained over a pressure range of 200 to −400 daPa at 226 Hz using a GSI tympanometer (Tympstar, Grason‐Stadler Inc., Denmark). The passing criterion for tympanograms was a type A curve with a peak within the range of –100 to +50 daPa and a static admittance of 0.3–1.6 mho.

### 2.3 Tinnitus Matching

The loudness and pitch of tinnitus were matched using a Tinnilogic BTD02 audiometer (Betterlife Medical Co., Ltd., Jiangsu, China) in a soundproof room. Participants were instructed to focus on the predominant pitch of their tinnitus. The tester then adjusted the frequency and intensity of the tonal stimulus until the participant confirmed that the external sound matched the pitch and loudness of the tinnitus percept. In cases of severe hearing loss in the tinnitus ear, the external sound was presented to the contralateral ear.

### 2.4 Measurement of Tinnitus Severity

The 25‐item beta version of the Tinnitus Handicap Inventory (THI) was used to subjectively assess the level of handicap experienced due to tinnitus (Newman et al. 1998; Newman et al. 1996). Participants were instructed to respond to each item on the inventory with “yes” (4 points), “sometimes” (2 points), or “no” (0 points). Based on the total score, the handicap caused by tinnitus was classified into one of five categories: slight, mild, moderate, severe, or catastrophic.

### 2.5 Group Classification

Twenty‐seven patients with THI scores of 36 points or lower were classified into the NBT group, while 21 patients with tinnitus scores higher than 36 points were assigned to the BT group, in accordance with the multidisciplinary European guideline for tinnitus (Cima et al. 2019). Additionally, 21 participants without tinnitus, matched for age, sex, and hearing threshold were selected as the healthy control group (HC group).

### 2.6 EEG Recording and Preprocessing

EEG recordings were conducted continuously while participants were seated in a soundproof room using a 256‐channel HydroCel Geodesic Sensor Net (GSN) from Electrical Geodesics Inc. (EGI). All electrode‐skin impedance values were maintained below 50 kΩ. EEG data were acquired at a sampling rate of 1000 Hz, with recordings performed at the same time of day for each participant. A 5‐min resting‐state EEG recording was obtained while participants sat in an armchair with their eyes closed. During the recordings, participants were instructed to remain awake, avoid eye movements or posture changes, and the participant and EEG were monitored for signs of drowsiness.

EEG data preprocessing was conducted using EEGLAB toolbox (Delorme and Makeig 2004) and custom scripts written in MATLAB2014a (The Mathworks, Natick, MA), as described in our previous study (Zhang et al. 2020). The resting state data were bandpass‐filtered between 0.5 and 150 Hz and notch‐filtered at 50 Hz. The signal from each trial was resampled at 500 Hz and segmented into 150 epochs of 2 s each. Data quality was visually inspected, and noisy channels or epochs with poor signal quality were either interpolated or removed. Artifacts such as eye movements, muscle movements, and heartbeats were manually identified and rejected.

Following preprocessing, the independent component analysis (ICA) was applied to the EEG data. ICA is a computational method used to solve a system of linear equations and is particularly effective for blind source separation. By decomposing the raw EEG data into independent components, ICA allows for the identification and separation of artifact‐related components (e.g., blinks, muscle activity, heartbeats) from neural activity‐related components. Artifact‐related components were removed, and the remaining components were recombined to reconstruct clean EEG signals, effectively eliminating artifacts while preserving genuine neural activity.

### 2.7 EEG Analysis

#### 2.7.1 Source Localization

EEG data source localization analysis was conducted using the EEGLAB toolbox and custom scripts written in MATLAB2014a, and Fieldtrip toolbox (Oostenveld et al. 2010). Preprocessed EEG data were converted into Fieldtrip format. A forward solution was constructed by loading the default head mold standard in Fieldtrip BEM using a single dataset. The noise was regressed in the time dimension for each trial, and the inverse solution was computed using the minimum‐norm estimate. Following source reconstruction, data from each trial were interpolated, smoothed, and aligned with the automated anatomical labeling (AAL) template to generate a time series for each brain region. This allowed for the modeling of source activations for individual trials. For functional connectivity analysis, source reconstruction was conducted in the frequency dimension.

#### 2.7.2 Region of Interest Analysis (ROI)

A region of interest (ROI)‐based approach was utilized, with ROI selection informed by previous studies on brain regions associated with the pathophysiology of tinnitus. The ROIs examined in this study encompassed the left and right Heschl gyrus, superior, middle, and inferior temporal cortex, superior, middle, and inferior orbitofrontal cortex, anterior and posterior cingulate cortex, parahippocampal gyrus, and insula.

#### 2.7.3 Phase–Amplitude Coupling (PAC) and Amplitude–Amplitude Coupling (AAC)

We calculated PAC at both sensor and source signal levels using the Kullback–Leibler‐based modulation index (KL‐MI). The analysis began with signal filtering for phase and amplitude estimation. For phase estimation, the original signals were filtered using a series of narrow‐band filters, ranging from 1 to 7 Hz in 2 Hz steps, with a 2 Hz bandwidth. For amplitude estimation, filtering was performed across a broader range from 7 to 150 Hz, also in 2 Hz steps with a 2 Hz bandwidth. Finite impulse response (FIR) filters were used for this purpose, utilizing the *eegfilt.m* function in EEGLAB with the FIR1 parameter setting. Following filtering, a Hilbert transform was applied to extract the instantaneous phase and amplitude separately. PAC calculations were restricted to phase‐amplitude pairs where the phase frequency was lower than the lower cut‐off frequency of the amplitude signals. For each phase‐amplitude pair, phases were sorted into 18 bins (20° bin width), and the corresponding amplitudes were averaged according to each phase bin. The amplitude‐phase distribution was then compared against a uniform distribution using KL‐MI to quantify PAC. The data were visualized in comodulograms, which represent KL‐MI values for different frequency pairs within each component pair or each sensor signal. Additionally, a single KL‐MI value was computed for each channel (or region) by averaging the KL‐MI values across the a priori defined phase frequency range of 4–8 Hz and amplitude–frequency ranges of 13–30 Hz and 30–70 Hz. A single PAC value for each ROI per subject was used for group comparisons.

AAC was calculated as previously described (Masimore et al. 2004). Briefly, AAC was calculated from the power spectra by segmenting each voxel time series into 2‐s epochs, which were then Fourier transformed using a Hanning window. AAC between high and low frequencies was then computed by correlating the squared moduli of the corresponding Fourier coefficients across segments. The analysis covered frequencies between 2 and 70 Hz with a step size of 0.5 Hz.

#### 2.7.4 Power–Power Coupling Calculation

The field potential data were initially transformed into frequency space using a fast Fourier transform (FFT). Power spectral information was extracted and normalized within each frequency band relative to the corresponding band's total power. Subsequently, the power values for the theta, beta, and gamma bands were averaged across participants. Spearman's correlation analyses were performed to examine the relationships between theta and beta power, as well as between theta and gamma power.

#### 2.7.5 Functional Connectivity

Functional connectivity analysis was performed between brain regions using frequency source reconstruction results. For each voxel, source signals were calculated retrospectively, followed by the calculation of coherence, phase lag index (PLI), phase locking value (PLV), and weighted phase lag index (wPLI) values. Regional functional connectivity values were then extracted according to the AAL116 template, and the ROIs × ROIs functional connectivity matrix was generated based on the predefined ROIs. False discovery rate (FDR) correction was applied for multiple comparisons.

#### 2.7.6 Machine Learning

K‐nearest neighbor (KNN) algorithm was employed to evaluate the classification performance of various features in distinguishing between the BT and NBT groups within the tinnitus cohort. The feature set incorporated power spectra, functional connectivity, and KL‐MI of theta–beta and theta–gamma couplings at both sensor and source levels. Given the limited sample size, leave‐one‐out cross‐validation was implemented to assess the model's performance. Feature dimensionality reduction was performed using Fisher's linear discriminant. To investigate the potential contribution of PAC in improving classification, we systematically examined different feature combinations followed by dimensionality reduction. The primary evaluation metric for classification performance was accuracy.

#### 2.7.7 Statistical Analysis

The normality of all measures was assessed using the Shapiro–Wilk test. Continuous variables were expressed as mean ± standard deviation (SD) for normally distributed data or median and interquartile range (IQR) for non‐normally distributed data. Between‐group differences in KL‐MI of PAC at the source level were examined using repeated‐measures ANOVA with an (ROI) × (Group) design, where ROI is the within‐subject factor and Group is the between‐subjects factor. For multiple comparisons correction, we applied the Bonferroni method, with post hoc tests using Bootstrap (1000 times) combined with FDR correction for *p*‐values. All analyses were performed using MATLAB2014a (The Mathworks, Natick, MA).

## 3 Results

The study included 21 BT patients (mean age of 44.31 ± 10.99 years, 52.63% male), 27 NBT patients (mean age of 38.74 ± 12.62 years, 42.31% male), and 21 HCs (mean age of 39.11 ± 14.70 years, 52.38% male). Demographic analysis revealed no significant group differences in age distribution (NBT vs. HC: *p* = 0.892; BT vs. HC: *p* = 0.062) or gender distribution (NBT vs. HC: *p* = 0.136; BT vs. HC: *p* = 0.754) (Table 1).

**TABLE 1:** Demographic characteristics of subjects in tinnitus and control group.

Variable	BT (*n* = 21)	NBT (*n* = 27)	HC (*n* = 21)
Age, median [IQR], years	45[37–51]	38[27–50]	36[28–45]
Gender, male, %	10 (52.63)	11 (42.31)	11 (52.38)
Duration, mean ± SD, years	3.47 ± 3.69	1.29 ± 2.05	NA
THI scores, mean ± SD	44.43 ± 9.32	18.52 ± 11.22	NA
Tinnitus matching			
Frequency, mean ± SD, kHz	5.17 ± 3.16	5.57 ± 3.14	NA
Loudness, dB	39.24 ± 14.74	40.30 ± 18.02	NA

*Note*: The number of asterisks indicates statistical significance against the HC. *P* values of continuous variables were assessed using the ANOVA and Bonferroni's method for post hoc tests or Mann‐Whitney *U* test.

Abbreviations: BT, bothersome tinnitus group; HC: healthy control group; IQR: interquartile range; NA: not applicable; NBT: non‐bothersome tinnitus group; PTA: pure‐tone average.

We compared the average thresholds at all frequencies among groups (Table 2). While no significant differences were observed at 2, 4, and 8 kHz, statistically significant differences emerged at 250, 500, and 1 kHz. However, thresholds at 250, 500, and 1 kHz. Notably, all measured thresholds at these frequencies remained below 25 dB HL across all groups, indicating that all subjects exhibited hearing within the normal range.

**TABLE 2:** The hearing thresholds of subjects in tinnitus and control group.

Frequency	NBT	BT	CT	*P* value		
				BT vs. NBT	BT vs. CT	NBT vs. CT
250 Hz	10.94 ± 8.55	14.44 ± 8.55	2.14 ± 5.44	0.001	0.000	0.003
500 Hz	9.80 ± 7.07	12.22 ± 6.47	4.89 ± 5.94	0.256	0.001	0.014
1000 Hz	9.90 ± 6.99	12.50 ± 7.28	6.19 ± 5.95	0.245	0.005	0.059
2000 Hz	10.97 ± 9.27	14.17 ± 8.09	9.76 ± 5.85	0.231	0.064	0.592
4000 Hz	15.10 ± 13.29	21.11 ± 13.37	16.55 ± 9.83	0.150	0.229	0.679
8000 Hz	14.23 ± 18.85	21.94 ± 18.76	22.22 ± 10.93	0.189	0.802	0.079

### 3.1 EEG Power

**FIGURE 2:**
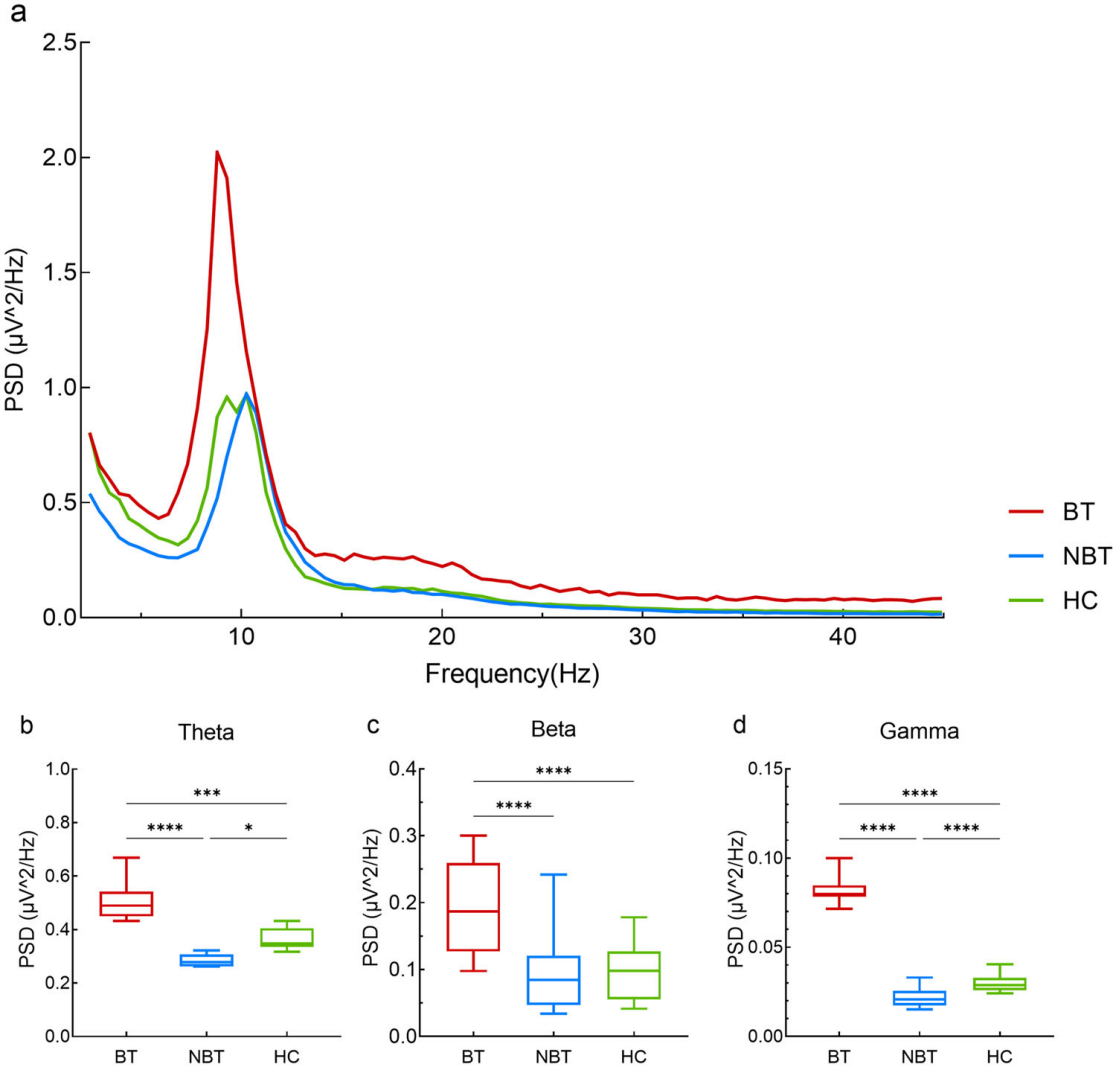
Power spectral density (PSD) of temporal cortex in the bothersome tinnitus (BT), non‐bothersome tinnitus (NBT), and healthy control (HC) groups. (a) Power of temporal cortex across three groups. The BT group showed a low‐frequency shift. (b) Theta power across three groups. (c) Beta power across three groups. (d) Gamma power across three groups. Theta, beta, and gamma power decreased in BT group compared to the HC group. There were no significant differences between the NBT and HC groups. (*p < 0.05; **p < 0.01; ***p < 0.001; ****p < 0.0001).

Power spectrum densities (PSDs) analysis was conducted by averaging signals across all temporal cortex electrodes within each group, revealing frequency‐specific activity patterns from delta to gamma bands (Figure 2). Compared to HCs, both tinnitus groups exhibited significant power differences in specific frequency bands: theta (*F* = 14.278, *p* < 0.001), beta (*F* = 39.478, *p* < 0.0001), and gamma (*F* = 1126.815, *p* < 0.0001). The BT group demonstrated significantly increased power across all three bands relative to HCs (theta: *p* < 0.0001, beta: *p* < 0.0001, gamma: *p* < 0.0001). In contrast, the NBT group showed reduced power in both theta (*p* = 0.035) and gamma (*p* < 0.0001) bands compared to HCs.

### 3.2 Power–Power Coupling at Sensor and Source Levels

Analysis of power–power cross‐frequency coupling revealed no significant differences among the BT, NBT, and HC groups (Figure S1).

### 3.3 Phase–Amplitude Coupling at Sensor and Source Levels

Our analysis of theta–beta and theta–gamma PAC across 26 ROIs at the source level revealed significant enhancements in tinnitus patients, particularly in the BT group compared to HCs. These enhancements were observed in both auditory and non‐auditory cortical regions, including the cingulate gyrus, insular cortex, frontal cortex, and parahippocampal gyrus (Figure S2).

**FIGURE 3:**
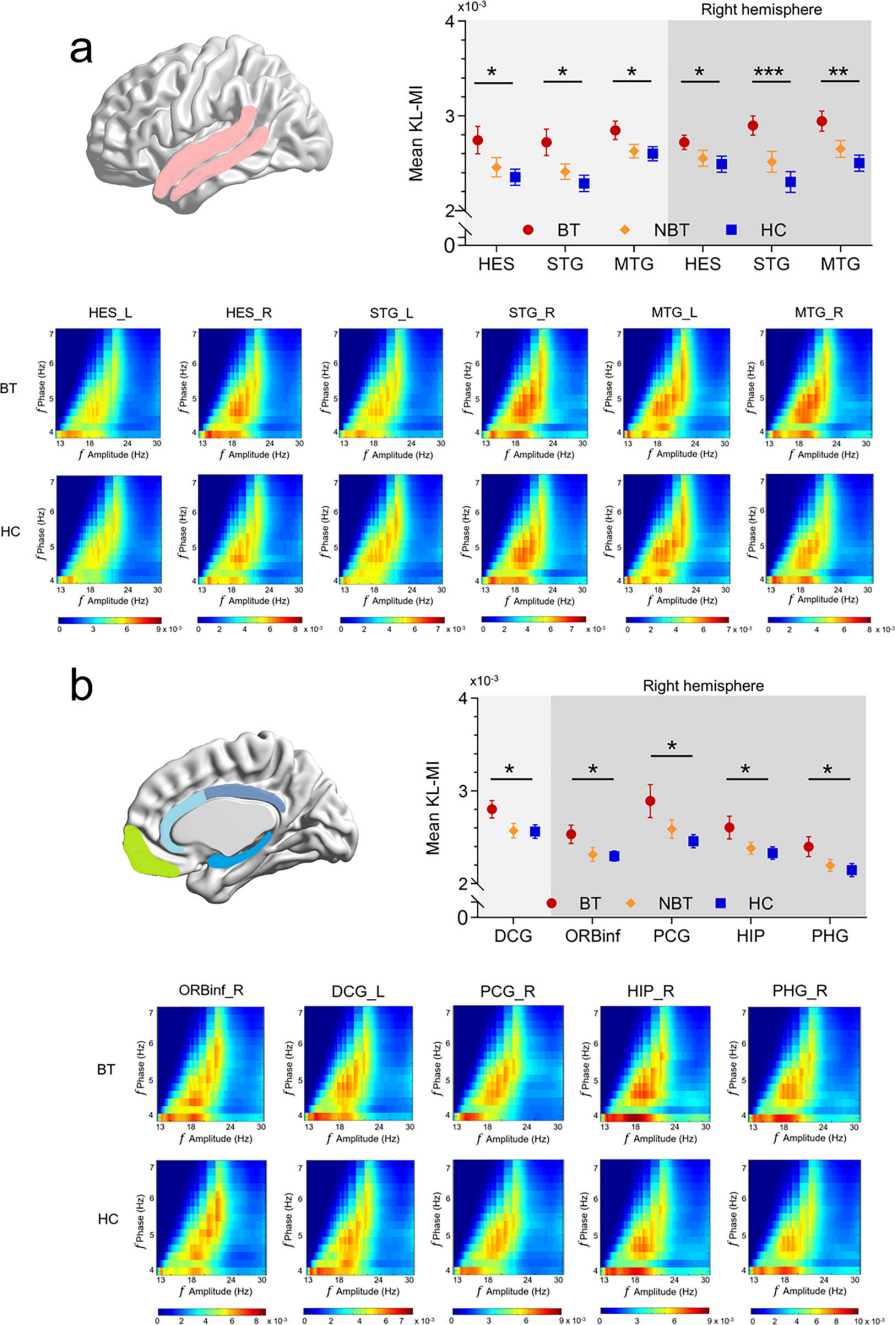
Theta–beta PAC observed at source levels in bothersome tinnitus (BT) compared to healthy controls (HC). (a) Analysis of PAC distribution across auditory cortex ROIs. Left: Regions marked show significant differences between patients and HC after FDR correction. Right: PAC of the six auditory cortex ROIs identify significant differences between BT and HC groups (error bars represent the standard errors of the means). Bottom: Group comodulograms showing the median of single KL‐MI across subjects in BT and HC groups in the six auditory cortex ROIs. (b) Analysis of PAC distribution across non‐auditory cortex ROIs comprising frontal cortex, cingulate gyrus, hippocampus, and hippocampus. Left: Regions marked showed significant differences between BT and NBTs versus HC groups after FDR correction. Right: PAC of the five non‐auditory cortex ROIs identify significant differences between BT and HC groups (error bars represent the standard errors of the means). Bottom: Group comodulograms showing the median of single KL‐MI across subjects in BT and HC controls in the five non‐auditory cortex ROIs. (*p < 0.05; **p < 0.01; ***p < 0.001; and ****p < 0.0001).

A significant ROI × Group interaction effect was identified for theta–beta PAC differences between the BT group and HCs (*F* = 2.419, *p* < 0.0001). Post hoc analysis demonstrated enhanced PAC in eleven specific ROIs within the BT group: (1) bilateral Heschl gyrus (HES, left: *F* = 5.388, *p* = 0.025; right: *F* = 4.128; *p* = 0.049), (2) bilateral superior temporal gyrus (STG, left: *F* = 6.968, *p* = 0.012; right *F* = 15.932, *p* < 0.0001), (3) bilateral middle temporal gyrus (STG, left *F* = 4.073, *p* = 0.048; right *F* = 10.603, *p* = 0.002), (4) right inferior orbitofrontal cortex (ORBinf, *Z* = 4.293, *p* = 0.045), (5) left dorsal anterior cingulate gyrus (DCG, *F* = 4.088, *p* = 0.047), (6) right posterior cingulate gyrus (PCG, *F* = 5.121, *p* = 0.029), (7) right hippocampus (HIP, *F* = 3.88, *p* = 0.050), and (8) right parahippocampus (PHG, *F* = 3.88, *p* = 0.050) (Figure 3). While the NBT group showed higher theta–beta PAC compared to HCs, no ROI × group significant interaction effect was observed (*F* = 0.177, *p* = 0.676).

**FIGURE 4:**
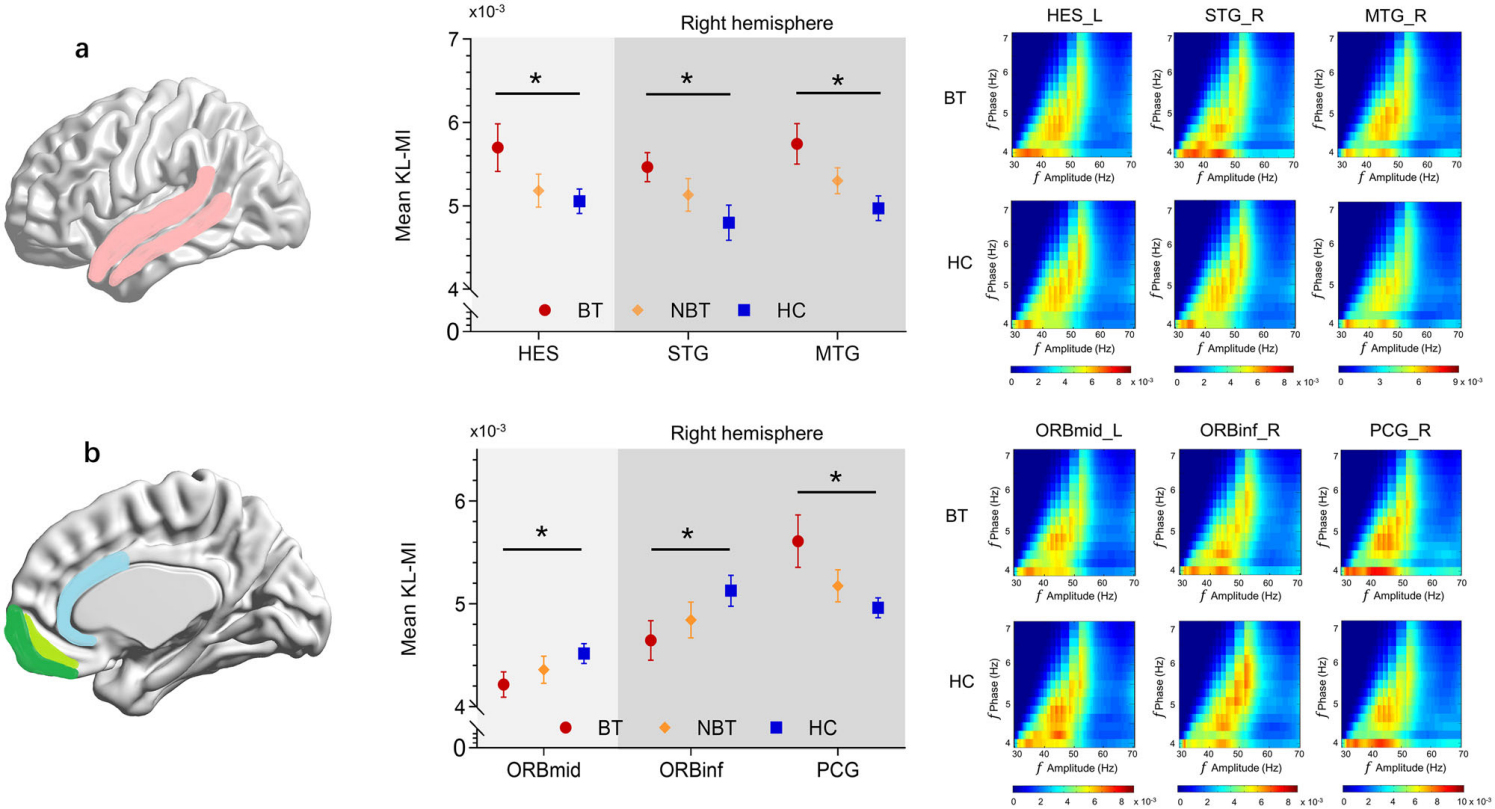
Theta–gamma PAC observed at source levels in bothersome tinnitus (BT) compared to the HC. (a) Analysis of PAC distribution across auditory cortex ROIs. Left: Regions marked showed significant differences between patients and controls after FDR correction. Right: PAC of the three auditory cortex ROI revealed significant differences between BT and HC groups (error bars represent the standard errors of the means). Bottom: Group comodulograms showing the median of single KL‐MI across subjects in BT and HC groups in the three auditory cortex ROIs. (b) Analysis of PAC distribution across non‐auditory cortex ROIs comprising frontal cortex and cingulate gyrus. Left: Regions marked showed significant differences between BT and HC groups. Right: PAC of the three non‐auditory cortex ROIs revealed significant differences between BT and HC groups (error bars represent the standard errors of the means). Bottom: Group comodulograms showing the median of single KL‐MI across subjects in BT and HC groups in the three non‐auditory cortex ROIs. (*p < 0.05; **p < 0.01 ***p < 0.001; and ****p < 0.0001).

A significant ROI × group interaction effect was observed for theta–gamma PAC differences between the BT and HC groups (*F* = 3.783, *p* < 0.0001). Post hoc analysis identified six ROIs with altered PAC in the BT group. Increased theta–gamma PAC was detected in: (1) left Heschl gyrus (HES, *F* = 4.019, *p* = 0.050), (2) right superior temporal gyrus (STG, *F* = 5.952, *p* = 0.019), (3) right middle temporal gyrus (MTG, *F* = 7.407, *p* = 0.010), (4) right posterior cingulate gyrus (PCG, *F* = 5.679, *p* = 0.022). Decreased theta–gamma PAC was observed in: (1) left middle orbitofrontal cortex (ORBmid, *F* = 3.725, *p* = 0.050), (2) right inferior orbitofrontal cortex (ORBinf, *F* = 3.941, *p* = 0.050) (Figure 4). While the NBT group exhibited generally higher theta–gamma PAC compared to the HC group (except in the orbitofrontal cortex), no significant interaction effect was found (*F* = 0.076, *p* = 0.784).

At the sensor level, analysis of signals recorded from F3, F4, Fz, T7, T8, Cz, and Pz electrodes revealed no significant differences in PAC among BT, NBT, and HC groups (Figure S3).

### 3.4 Relationship Between PAC and Other Features

**FIGURE 5:**
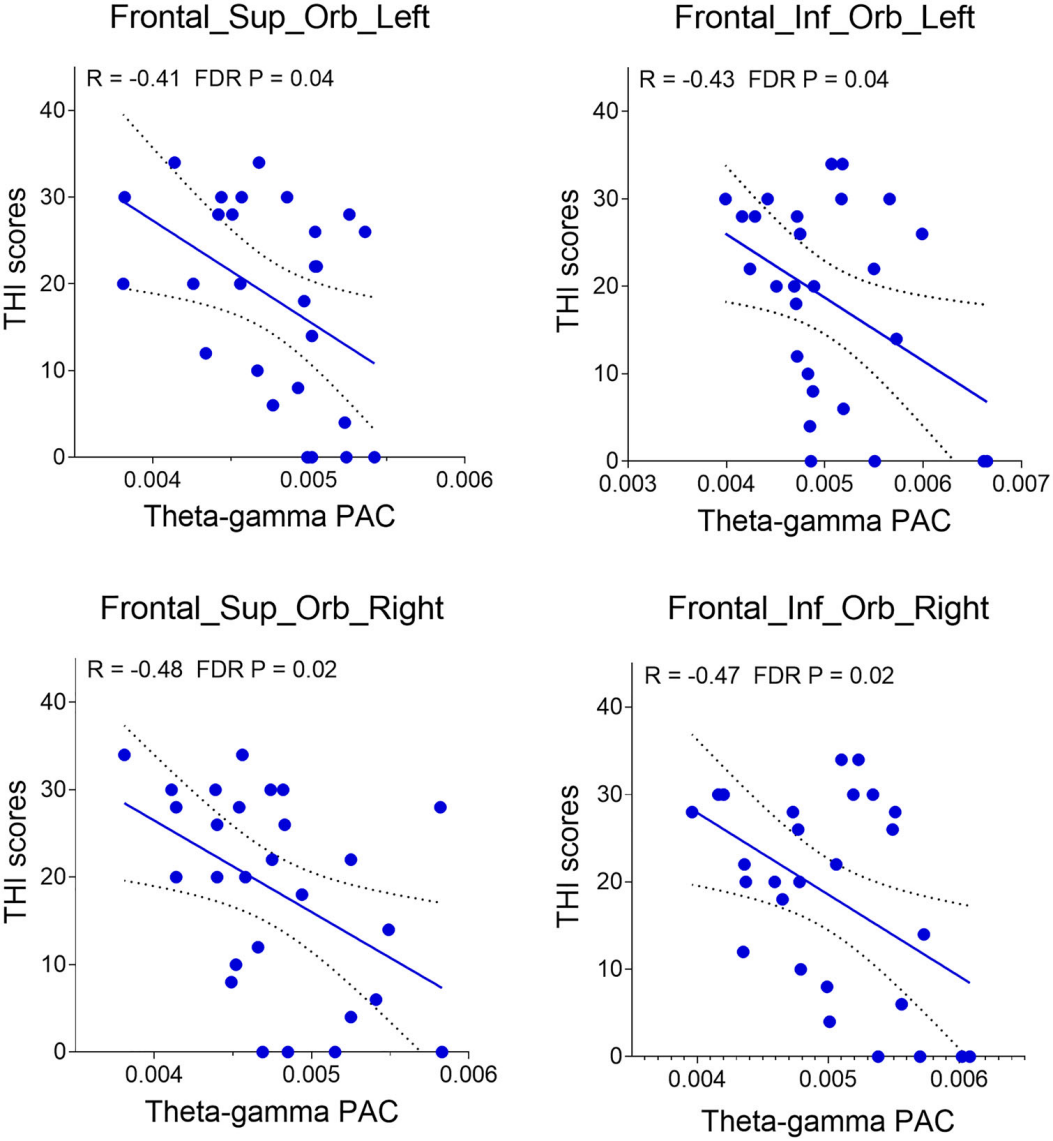
Correlation between theta–gamma PAC and THI scores in non‐bothersome tinnitus (NBT) group. A significant negative correlation was observed between THI scores and theta–gamma PAC in bilateral ORBsup and ORBinf cortices, whereas no significant correlations were observed in the bothersome tinnitus (BT) group.

We analyzed the correlations between theta–beta and theta–gamma PAC and clinical characteristics in tinnitus patients. Post‐hoc linear regression analyses revealed significant negative correlations between THI scores and theta–gamma PAC at the source level in NBT participants. Specifically, these correlations were observed in bilateral ORBsup (left: *r* = −0.41, *p* = 0.04; right: *r* = −0.48, *p* = 0.02) and bilateral ORBinf (left: *r* = −0.43, *p* = 0.04; right: *r* = −0.47, *p* = 0.02) (Figure 5). In contrast, BT subjects showed no significant relationships between theta–beta/gamma PAC and tinnitus characteristics (all *p* > 0.05).

### 3.5 Machine Learning

We initially developed classification models, respectively, using all sensor‐level and source‐level features. The sensor‐level model achieved moderate performance (accuracy: 0.75; sensitivity: 0.70; and specificity: 0.81), while the source‐level model demonstrated superior classification accuracy (accuracy: 0.91; sensitivity: 0.89; and specificity: 0.95), suggesting that source‐level features contain more discriminative information for distinguishing between BT and NBT.

Using purely functional connectivity features yielded an average classification accuracy of 89.58% for differentiating BT from NBT. Interestingly, integrating power and functional connectivity features did not improve model accuracy, indicating that the power features may introduce noise. However, integrating functional connectivity features and KL‐MI of theta–beta and theta–gamma features across all ROIs resulted in optimal classification performance (accuracy = 91.67%), with a true positive rate of 0.89 and a true negative rate of 0.95. These findings demonstrate that KL‐MI measures of PAC provide valuable discriminative information along specific feature dimensions.

## 4 Dicussions

Our findings revealed distinct patterns of PAC alterations in tinnitus patients.

BT patients exhibited enhanced theta–beta PAC in multiple regions, including the auditory cortex, anterior and posterior cingulate gyrus, inferior orbitofrontal cortex, and parahippocampal gyrus, compared to HCs. Similarly, theta–gamma PAC was increased in the auditory cortex and post cingulate gyrus. Notably, we observed attenuated theta–gamma PAC in the orbitofrontal cortex of BT patients. In the NBT group, PAC in the orbitofrontal cortex showed negative correlations with THI scores, indicating its involvement in tinnitus perception and associated emotions.

To our knowledge, we first developed a method to objectively discriminate between BT and NBT subjects using EEG‐derived metrics related to TCD and noise‐canceling mechanisms. Our approach combined the assessment of theta–beta/gamma PAC in limbic‐auditory networks with KNN classification, achieving a maximum classification accuracy of 91.67%. This optimal performance was attained by incorporating theta–beta and theta–gamma PAC measures across all ROIs. The identified PAC patterns—characterized by enhanced theta–beta/gamma PAC in the auditory‐limbic regions and reduced theta–gamma PAC in the orbitofrontal cortex—may serve as objective biomarkers to distinguish subjects with BT from those with NBT and HC. These findings provide new insights into the neural mechanisms underlying different tinnitus subtypes.

### 4.1 The Limbic System and Tinnitus

Previous neuroimaging studies have consistently implicated the limbic system and prefrontal cortex as key dysfunctional nodes underlying tinnitus‐related annoyance. The limbic system comprises three distinct but overlapping networks (Catani et al. 2013): (1) the hippocampal and parahippocampal network, (2) the temporal‐amygdala‐orbitofrontal network, and (3) the default‐mode network. These networks collectively mediate crucial cognitive‐emotional processes, including memory formation and the integration of emotional states with cognition and behavior. Notably, robust anatomical connections exist between the auditory and limbic systems. These connections include direct links between the hippocampus and auditory association cortices via the parahippocampal cortex and indirect routes through forebrain pathways such as the medial frontal cortex, insula, and amygdala (Kraus and Canlon 2012).

Emerging evidence suggests that functional interactions between limbic structures and auditory cortices play a crucial role in mediating the emotions associated with tinnitus phantom (Rauschecker et al. 2015; Kraus and Canlon 2012; Mühlau et al. 2006; Landgrebe et al. 2009; Mayberg et al. 2005; Vanneste et al. 2010; Landgrebe et al. 2009; Mirz et al. 2000; Hofmeier et al. 2018). This is supported by observed enhancements in connectivity between auditory cortical regions and limbic emotional centers (Vanneste et al. 2010). Structural neuroimaging studies have revealed consistent gray matter volume reductions in hippocampal and subcallosal structures among tinnitus patients (Mühlau et al. 2006; Landgrebe et al. 2009), with spatial patterns overlapping those observed in chronic pain populations (Rauschecker et al. 2015). Furthermore, the functional abnormalities in the anterior cingulate and medial prefrontal cortex of tinnitus patients resemble the neural signatures characteristic of depressive disorders (Mayberg et al. 2005). Moreover, the limbic system may serve a dual role in tinnitus pathophysiology: not only does it contribute to the emotional distress associated with tinnitus, but it may also be directly involved in modulating the phantom perception itself (Rauschecker et al. 2010).

### 4.2 Prefrontal Cortex Involved in Negative Emotion Related Diseases

Our study revealed distinct EEG characteristics between BT and NBT. While both groups exhibited increased temporal cortex activity, BT patients demonstrated significantly reduced prefrontal cortex activities. This finding aligns with growing evidence suggesting a bidirectional relationship between tinnitus and depression, with the prefrontal cortex emerging as a potential neural hub in tinnitus pathophysiology. Neuroimaging studies confirmed the existence of neural circuits that are activated both in depression and tinnitus (Langguth et al. 2011). Clinically, transcranial magnetic therapy of the prefrontal cortex, commonly used in depression, has also obtained good results in patients with tinnitus, with higher efficacy than the traditional temporoparietal cortex (Wang et al. 2015). Animal studies also support this connection: optogenetic activation of the amygdala suppresses spontaneous auditory cortex activity (Aizenberg et al. 2019) while striatal electrical stimulation reduces the spontaneous firing rate of neurons in the auditory cortex and reduces the tinnitus‐like behavior (Wang et al. 2021). Therefore, the affective disorders interact with tinnitus or may also just be a consequence of tinnitus (Leaver et al. 2011). The mechanism between tinnitus and affective disorders is still not clear.

Besides the auditory cortex, we found that TCD oscillations extend to the frontal cortex, a key neural substrate in tinnitus pathophysiology. Notably, the theta–gamma coupling was significantly attenuated in the frontal cortex in the BT group compared to the NBT and HC groups. Furthermore, we observed a negative correlation between the orbitofrontal cortex PAC and THI scores in BT patients. These findings suggest two potential interpretations: One hypothesis is that the disrupted PAC in BT patients' frontal cortex may be indicative of defective noise cancellation, potentially contributing to the emergence of troublesome tinnitus. Second, the stronger frontal PAC in NBT patients compared to the BT patients (though weaker than in HCs) may explain why NBT patients perceive tinnitus as less bothersome. This pattern could indicate partially preserved noise‐cancellation functionality in NBT patients. However, due to the interaction between tinnitus and negative emotion not being understood, the attenuated prefrontal TCD activities could be the consequence rather than a cause of chronic and distressing tinnitus. Further research is needed to elucidate the causal relationships between these neural alterations and tinnitus‐related distress.

### 4.3 Limitation and Future Directions

In our study, though the included subjects with clinically normal hearing thresholds, we acknowledge that some tinnitus patients may experience damage to the synaptic junction between cochlear hair cells and auditory nerve fibers resulting in a loss of synapses. This highlights the need for more rigorous auditory function matching between groups in future tinnitus research. Our EEG findings, interpreted in the context of TCD and noise‐cancellation models, provide an analytical framework that could be evaluated with a larger and more diverse group of tinnitus patients and controls. Although EEG offers excellent temporal resolution for assessing PAC coupling in different frequency bands, identifying the sources/structures underlying these measures can be problematic, especially for deeper structures. To address these limitations, multimodal neuroimaging approaches combining EEG with structural and functional MRI (fMRI) could provide complementary insights. Structural MRI could reveal gray and white matter differences in auditory and limbic structures across groups, potentially explaining observed EEG anomalies in BT and NBT patients. While fMRI offers a plethora of resting‐state functional connectivity metrics, its relatively poor temporal resolution compared to EEG could be offset by simultaneous EEG‐fMRI acquisition. Such integrated approaches may provide new insight into the functional coupling patterns underlying TCD and noise‐cancellation mechanisms in tinnitus pathophysiology.

## 5 Conclusion

Our study revealed distinct PAC patterns in BT patients, characterized by enhanced theta–beta/gamma PAC in auditory‐limbic regions and reduced theta–gamma PAC in the orbitofrontal cortex, which significantly negatively correlated with tinnitus distress. These PAC features could effectively differentiate between BT and NBT subjects by machine learning, highlighting the specificity of PAC for tinnitus.

## Author Contributions

**Ying Wang**: writing – original draft, methodology. **Xin Rong Ma**: writing ‐ review and editing, visualization. **Jiajia Zhang**: methodology, writing – original draft, data curation. **Xuan Huang**: software. **Shujian Huang**: investigation. **Yanmei Feng**: investigation. **Haibo Shi**: funding acquisition. **Hui Wang**: resources, project administration. **Richard Salvi**: writing – review and editing. **Shankai Yin**: funding acquisition.

## Ethics Statement

This study was approved by the Institutional Ethics Review Board of Shanghai, the Sixth People's Hospital affiliated with Shanghai Jiao Tong University (Approval number: 2020–122).

## Peer Review

The peer review history for this article is available at https://publons.com/publon/10.1002/brb3.70437

## Supporting information



Supporting Information

Supplementary Fig.1:Power to power cross‐frequency coupling across the three groups at sensor level and source level.

Supplementary Fig. 2:Radar plot illustrating presence of cross‐frequency coupling in the auditory cortex, cingulate cortex, insular cortex, frontal cortex, hippocampus, and para hippocampus gyrus for theta‐beta/gamma coupling.
Asterisks indicates if the PAC of BT and NBT is significantly different from HC after FDR correction (*: p< 0.05; ** p< 0.01 ***: p< 0.001 ****: p< 0.0001). Left: The figure demonstrates the presence of theta–beta coupling for bothersome tinnitus (BT, red), non‐bothersome tinnitus (NBT) (blue) and controls (green) in the auditory cortex and non‐auditory cortex. Right: The figure demonstrates the presence of theta–gamma coupling for (red), NBT (blue) and controls (green) in the auditory cortex and non‐auditory cortex.

Supplementary Fig. 3: Analysis of theta‐beta and theta‐gamma PAC at the sensor level.
Left: Electrodes F3, F4, Fz, T7, T8, Cz, Pz. Right: Box plot showing the theta‐beta and theta‐gamma PAC extracted from the seven electrodes of bothersome tinnitus (BT), non‐bothersome tinnitus (NBT) and HC groups. None of the differences reached statistical significance.

## Data Availability

The data that support the findings of this study are available from the corresponding author upon reasonable request.
