# Supplementary material for: Theta–Beta/Gamma Coupling Identifies Bothersome Tinnitus Induced by Thalamocortical Dysrhythmia
Source: Brain Behav. 2025 Jun 12;15(6):e70437. doi: 10.1002/brb3.70437 (PMC12159765; doi:10.1002/brb3.70437)
Supplement: Supplementary file 1 — Supporting Information [file BRB3-15-e70437-s001.docx]

**Supplementary Fig.1：**Power to power cross-frequency coupling across the three groups at sensor level and source level.

**Supplementary Fig. 2**：Radar plot illustrating presence of cross-frequency coupling in the auditory cortex, cingulate cortex, insular cortex, frontal cortex, hippocampus, and para hippocampus gyrus for theta-beta/gamma coupling.

Asterisks indicates if the PAC of BT and NBT is significantly different from HC after FDR correction (*: p<0.05; ** p<0.01 ***: p<0.001 ****: p<0.0001). Left: The figure demonstrates the presence of theta–beta coupling for bothersome tinnitus (BT, red), non-bothersome tinnitus (NBT) (blue) and controls (green) in the auditory cortex and non-auditory cortex. Right: The figure demonstrates the presence of theta–gamma coupling for (red), NBT (blue) and controls (green) in the auditory cortex and non-auditory cortex.

**Supplementary Fig. 3**: Analysis of theta-beta and theta-gamma PAC at the sensor level.

Left: Electrodes F3, F4, Fz, T7, T8, Cz, Pz. Right: Box plot showing the theta-beta and theta-gamma PAC extracted from the seven electrodes of bothersome tinnitus (BT), non-bothersome tinnitus (NBT) and HC groups. None of the differences reached statistical significance.
