# Supplementary figures and images for: Theta–Beta/Gamma Coupling Identifies Bothersome Tinnitus Induced by Thalamocortical Dysrhythmia
Source: Brain Behav. 2025 Jun 12;15(6):e70437. doi: 10.1002/brb3.70437 (PMC12159765; doi:10.1002/brb3.70437)

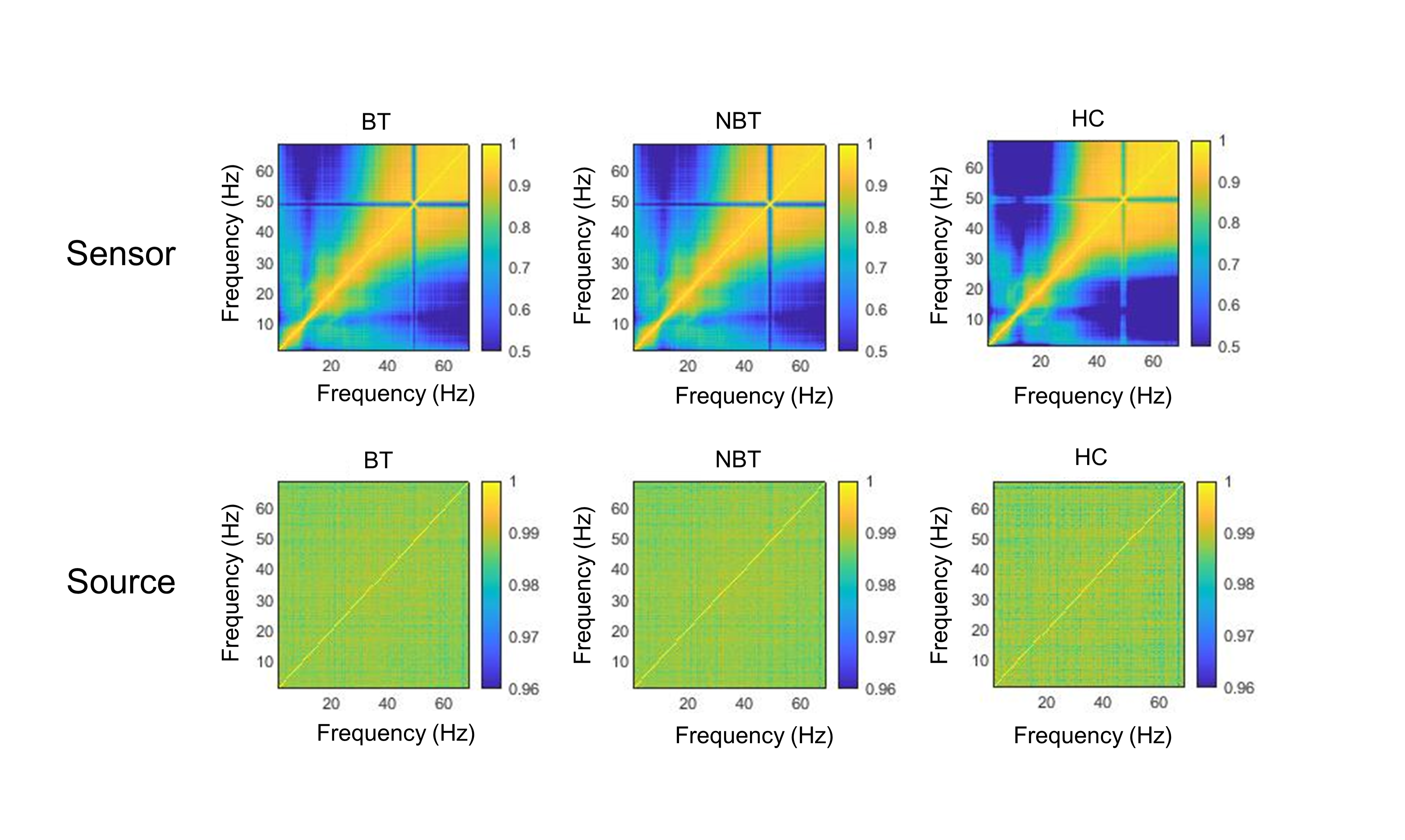

Supplement: Supplementary file 2 — Supplementary Fig.1:Power to power cross‐frequency coupling across the three groups at sensor level and source level. [file BRB3-15-e70437-s004.tif]

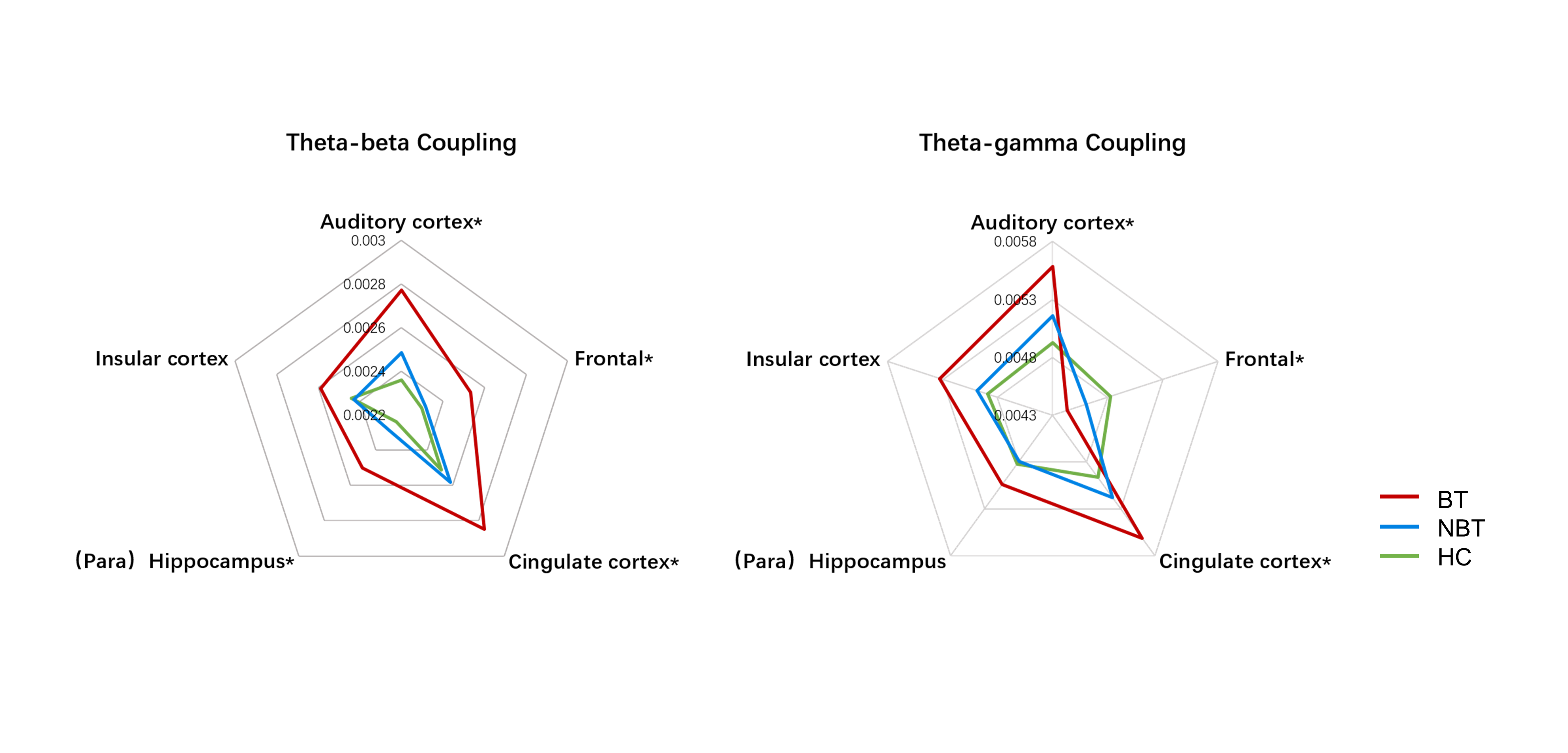

Supplement: Supplementary file 3 — Supplementary Fig. 2:Radar plot illustrating presence of cross‐frequency coupling in the auditory cortex, cingulate cortex, insular cortex, frontal cortex, hippocampus, and para hippocampus gyrus for theta‐beta/gamma coupling. Asterisks indicates if the PAC of BT and NBT is significantly different from HC after FDR correction (*: p< 0.05; ** p< 0.01 ***: p< 0.001 ****: p< 0.0001). Left: The figure demonstrates the presence of theta–beta coupling for bothersome tinnitus (BT, red), non‐bothersome tinnitus (NBT) (blue) and controls (green) in the auditory cortex and non‐auditory cortex. Right: The figure demonstrates the presence of theta–gamma coupling for (red), NBT (blue) and controls (green) in the auditory cortex and non‐auditory cortex. [file BRB3-15-e70437-s002.tif]

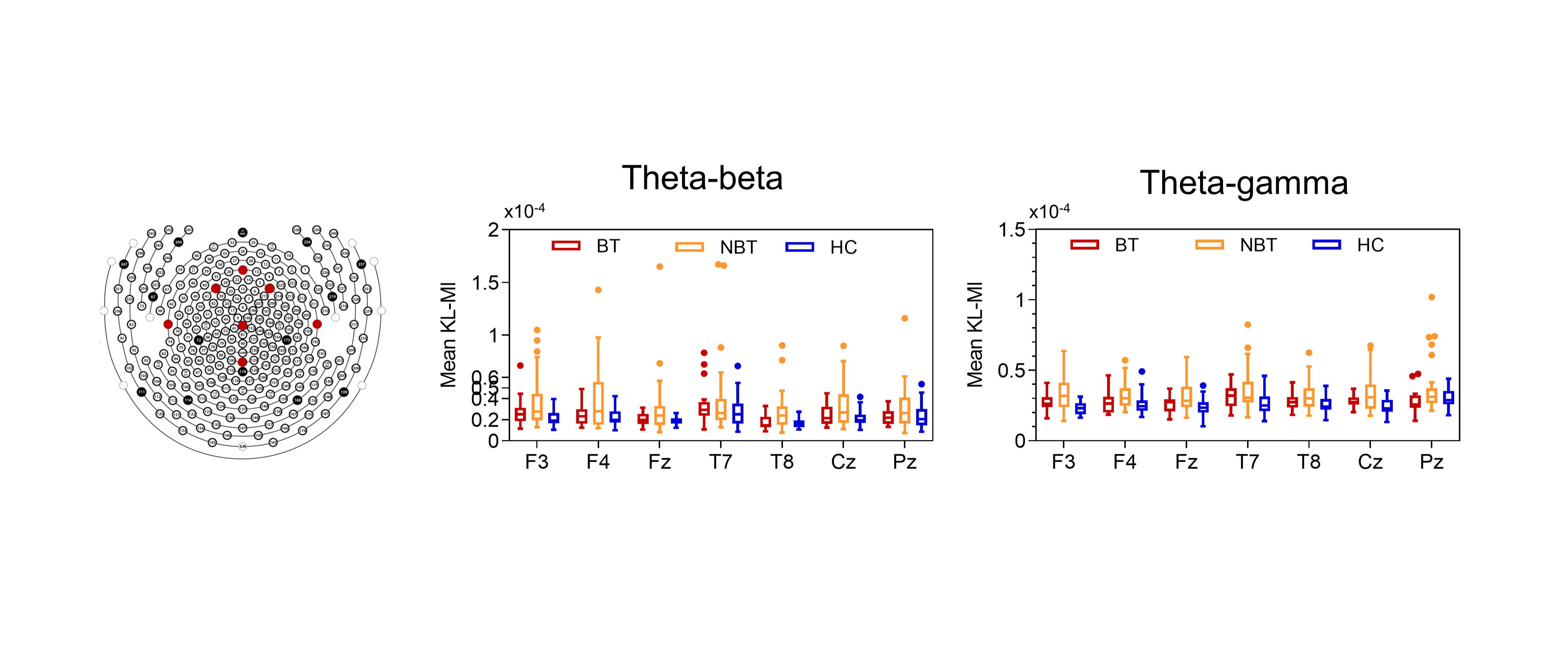

Supplement: Supplementary file 4 — Supplementary Fig. 3: Analysis of theta‐beta and theta‐gamma PAC at the sensor level. Left: Electrodes F3, F4, Fz, T7, T8, Cz, Pz. Right: Box plot showing the theta‐beta and theta‐gamma PAC extracted from the seven electrodes of bothersome tinnitus (BT), non‐bothersome tinnitus (NBT) and HC groups. None of the differences reached statistical significance. [file BRB3-15-e70437-s003.tif]
